# Response to pembrolizumab in a patient with primary lung adenocarcinoma originated from indium lung

**DOI:** 10.1186/s12890-021-01474-x

**Published:** 2021-03-31

**Authors:** Yasuharu Sekine, Hideo Ichimura, Sho Ueda, Keisuke Kobayashi, Takeshi Nawa, Atsuko Amata, Tatsuya Chonan, Akiko Sakata, Yoji Komatsu, Yukio Sato

**Affiliations:** 1 Department of Thoracic Surgery, Faculty of Medicine, Hitachi General Hospital, University of Tsukuba, Hitachi Medical Education and Research Center, 2-1-1 Jyounan, Hitachi, Ibaraki 317-0077 Japan; 2 Department of Thoracic Surgery, University of Tsukuba, 1-1-1 Tennodai, Tsukuba, Ibaraki 305-8575 Japan; 3 Hitachi Medical Education and Research Center, Faculty of Medicine, University of Tsukuba, 2-1-1 Jyounan, Hitachi, Ibaraki 317-0077 Japan; 4 Department of Respiratory Medicine, Hitachi General Hospital, 2-1-1 Jyounan, Hitachi, Ibaraki 317-0077 Japan; 5 Department of Medicine, Nikko Memorial Hospital, Hitachi, Ibaraki 317-0064 Japan; 6 Department of Pathology, Hitachi General Hospital, 2-1-1 Jyounan, Hitachi, Ibaraki 317-0077 Japan; 7 Department of Neurosurgery, Hitachi General Hospital, 2-1-1 Jyounan, Hitachi, Ibaraki 317-0077 Japan

**Keywords:** Indium-tin oxide, Carcinogenicity, Occupational lung disease, Lung cancer, Immune checkpoint inhibitor

## Abstract

**Background:**

Indium is a metal used as a compound called indium-tin oxide for liquid crystal display. Its inhalation causes lung toxicity, resulting in a new occupational lung disease called indium lung. Although the carcinogenicity of indium has been reported in an animal model, its carcinogenicity in humans is unknown.

**Case presentation:**

This is the first reported case of a primary lung cancer originating from indium lung. In this report, we describe a 46-year-old man with interstitial pneumonia-type indium lung diagnosed 16 years ago. The initial symptom was left chest pain, and computed tomography showed a mass adjacent to the aorta with left pleural effusion. Specimens collected using video-assisted thoracoscopy revealed an adenocarcinoma with a high expression of programmed cell death-ligand 1 (cT4N0M1a stage IVA). Although the lesions showed a remarkable aggressive nature, the patient benefited from pembrolizumab, a monoclonal antibody against programmed cell death 1, which was used as second-line therapy for 2 years.

**Conclusions:**

It is important for clinicians to be aware of lung cancer development in indium-exposed workers or in patients with indium lung, as this could have an aggressive behavior. Treatment with immune checkpoint inhibitors is an option even in patients with interstitial pneumonia-type indium lung.

## Background

Indium is a metal that can be used to create a compound called indium-tin oxide, which is used in liquid crystal displays. Its inhalation can cause an occupational lung disease called indium lung, which manifests as alveolar proteinosis, interstitial pneumonia or pulmonary fibrosis, and emphysema [1, 2]. In addition to lung toxicity, the carcinogenicity of indium has been demonstrated in rat models with a chronic exposure to indium [3]. Therefore, indium carcinogenicity in humans is of great concern. Although a cohort study of indium-exposed workers reported two lung cancer cases as incident cases, there is no information on the pathological type or therapeutic process [4]. This patient has been described twice in prior reports; first, as a case of indium lung [5], and second, as a case of lung cancer, which was the first pathologically confirmed case originating from indium lung [6]. In this report, we further describe the clinical characteristics of the disease and its detailed therapeutic course.

## Case presentation

A 46-year-old man who complained of left-sided chest pain for 2 weeks was transferred to our hospital for exploratory video-assisted thoracoscopy (VATS). He had a history of indium-tin oxide inhalation for 12 years, resulting from occupational exposure. He underwent chest X-ray at his workplace during routine check-up, and it showed a bilateral reticular shadow in the middle and lower lung fields, and he complained of cough and phlegm. He then underwent chest computed tomography (CT) and trans-bronchial lung biopsy, which led to the diagnosis of an indium lung (interstitial pneumonia or pulmonary fibrosis type) 16 years ago [5]. Subsequently, he was transferred to a section of his workplace where he was not exposed to indium. He went for a medical checkup twice a year. Six years before this consultation (10 years after his transfer to a non-indium section in his workplace), he had an acute exacerbation of indium lung, which was treated with steroid pulse therapy. After that, the patient received steroids, which were tapered off for 3 years. He was a non-smoker and also had a colon polyp. At the time he was diagnosed with indium lung, the serum indium levels (sIn) were 40.42 ng/mL (normal range: 0.06 ± 0.03 ng/mL) and serum Krebs von den Lungen-6 (KL-6, as a biomarker of interstitial pneumonia; normal range: < 500 U/mL) levels were 1930 U/mL. Although cessation of indium exposure gradually decreased sIn and KL-6 levels, they remained higher than normal (two years ago, sIn and KL-6 levels were 8.65 ng/mL and 1300 U/mL, respectively).

**Fig. 1:**
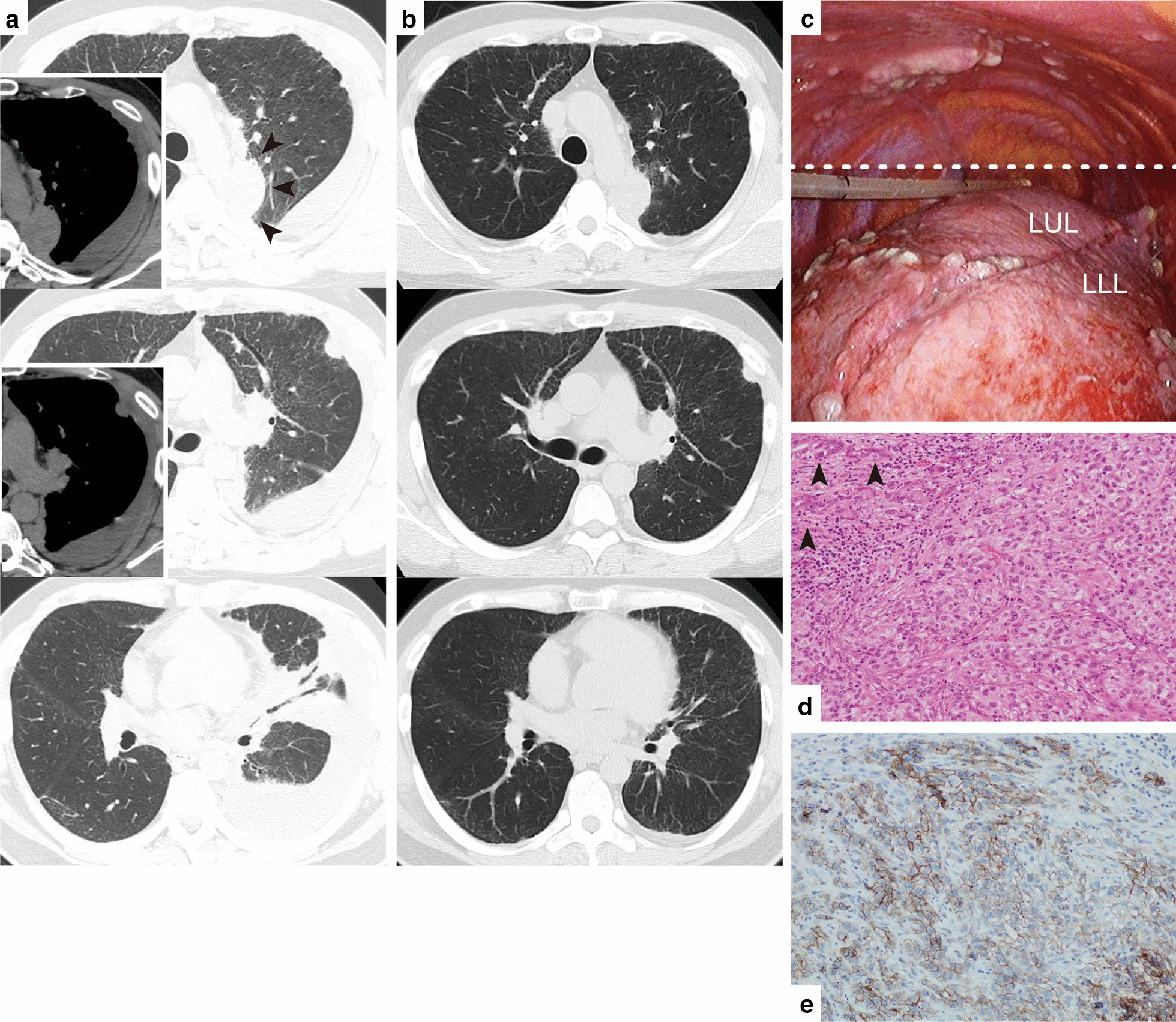
**a** Chest computed tomography (CT) at different planes taken before video-assisted thoracoscopic (VATS) biopsy. Arrowheads indicate a mass lesion adjacent to the aorta; **b** chest CT in the planes identical to those in **a** taken 12 days prior at the previous hospital; **c** combined photograph of intrathoracic finding taken during the VATS. A chest drain tube was placed. The dashed line indicates a combination of two intraoperative stills. *LUL* left upper lobe, *LLL* left lower lobe; **d** pathological image of the pleural nodule stained using hematoxylin–eosin (original magnification × 200). Arrowheads indicate acinar nests of tumor cells; **e** pathological image with immunohistochemical staining of PDL-1 (22C3) (original magnification × 200)

On admission, computed tomography (CT) showed pleural effusion, pleural nodules, and a mass (32 × 30 mm in size) adjacent to the aorta (Fig. 1a). A comparison with CT scans taken 12 days ago at the previous hospital (Fig. 1b) demonstrated a significant deterioration of the entire lesion. VATS under general anesthesia revealed that there were multiple nodules of various sizes on the visceral and parietal pleura of the left thoracic cavity (Fig. 1c). We performed biopsy of the pleural nodule on the diaphragm and wedge resection of the peripheral region of the left lower lobe, where it was in contact with the diaphragm. The postoperative course was uneventful, and the patient was transferred to the previous hospital on postoperative day 8.

Histopathological examinations revealed that the nodule consisted of polygonal tumor cells with nuclear atypia that formed solid nests partially, including acinar nests (Fig. 1d). On immunohistochemical examination, calretinin and D2-40 were negative, and thyroid transcription factor 1 (TTF-1) was positive in tumor cells within the acinar nest. Based on these findings, the patient was diagnosed with primary lung adenocarcinoma. The tumor cells did not show any driver mutations in *EGFR*, *ALK*, and *ROS1*. Immunohistochemical analysis of programmed cell death receptor ligand 1 (22C3) showed highly positive staining (Tumor Proportion Score > 95%) (Fig. 1e). In the lung parenchyma specimens, cholesterol granulomas and fibrotic changes in the alveolar septa were apparent [6].

**Fig. 2:**
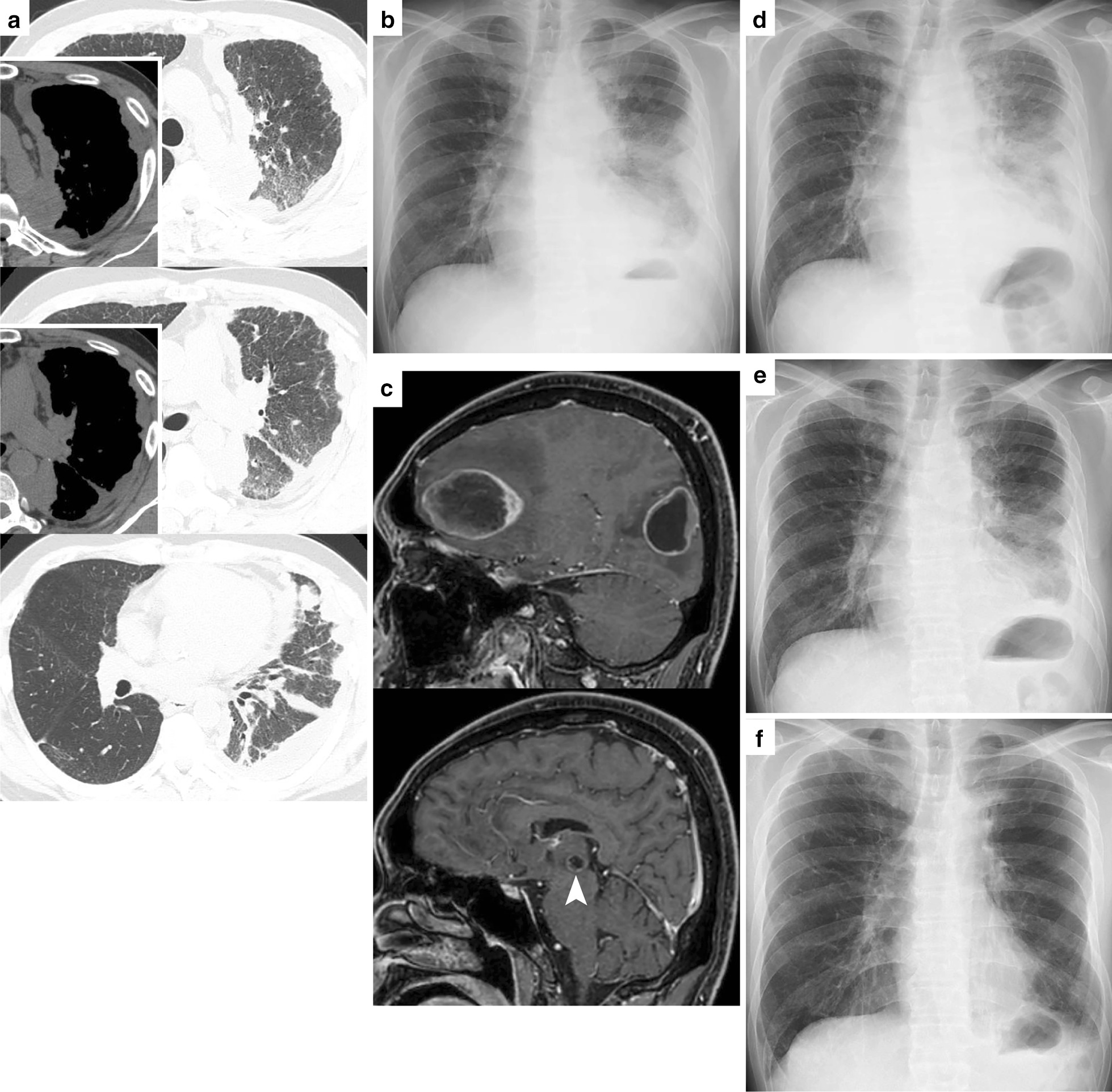
**a** Chest computed tomography in the planes identical to those in Fig. 1 taken 38 days after the video-assisted thoracoscopy; **b** chest X-ray taken on the day before the first pembrolizumab administration; **c** enhanced brain magnetic resonance imaging of the sagittal plane shows metastatic brain tumors. The arrowhead in the lower panel indicates the lesion in the right thalamus; **d** chest X-ray taken on day 30 of pembrolizumab administration; **e** chest X-ray taken on day 39 of pembrolizumab administration; **f** chest X-ray taken after 30 cycles of pembrolizumab

The patient was transferred to our hospital again for chemotherapy 37 days after the VATS. Pre-chemotherapy CT showed further growth of all the lesions in the left thoracic cavity (Fig. 2a). Fluorodeoxyglucose positron emission tomography and enhanced brain magnetic resonance imaging (MRI) showed no extra-thoracic metastasis. Therefore, the patient was diagnosed with stage IVA left lung cancer (cT4N0M1a). Considering his history of acute exacerbation of the indium lung, he received cytotoxic agents (a regimen of carboplatin and nanoparticle albumin-bound paclitaxel) as first-line therapy. This was because we were concerned about the adverse effects of the immune checkpoint inhibitor (ICI), especially ICI-related pneumonitis/interstitial lung disease [7, 8]. While the first-line treatment showed a transient response, a CT scan taken after four cycles of chemotherapy showed progressive disease with direct invasion of the sixth thoracic vertebra and metastasis of the fourth vertebra. Palliative radiotherapy (30 Gy) for the fourth and sixth thoracic vertebrae was administered, and pembrolizumab (200 mg/body) was administered as second-line therapy (Fig. 2b shows a chest X-ray taken one day before ICI initiation).

On day 10 of the first ICI administration, he visited a hospital with impaired consciousness and vertigo. Brain MRI showed multiple brain metastases measuring 5 cm in the left frontal lobe, 3.5 cm in the left occipital lobe, and 7 mm in the right thalamus (Fig. 2c). Complete resection of the tumor in the frontal lobe and incomplete resection of the tumor in the occipital lobe were performed. An MRI scan taken on postoperative day 6 showed an increase in the size of the lesion in the left occipital lobe and the right thalamus, and new small nodular lesions were seen in the frontal lobes. Therefore, whole-brain radiotherapy (WBRT) of 35 Gy with 17 fractions was initiated on postoperative day 9. Although no improvement was observed on the chest X-ray taken on day 30 of ICI administration (on day 5 of WBRT) (Fig. 2d), the chest X-ray taken on day 39 of ICI administration (2 weeks after WBRT initiation) showed an improved transparency of the left thorax (Fig. 2e), which we considered to be a response to pembrolizumab. Following this, we restarted pembrolizumab 72 days after the first administration.

The patient has received 36 cycles of pembrolizumab without any adverse events (Fig. 2f). He is visiting our outpatient clinic and conducting almost all activities of daily life after the diagnosis 30 months ago.

## Discussion and conclusions

Herein, we reported the first detailed case of primary lung adenocarcinoma that arose from indium lung. This cancer had a very aggressive nature, which was indicated by a rapid deterioration after onset and by its treatment process. The carcinogenicity of indium in this patient was further emphasized by the fact that he did not have a history of smoking.

We have clinically determined the lesion adjacent to the aorta that was intrapulmonary as the primary site. Although we could not confirm whether the lesion was intrapulmonary or extra-pulmonary during the VATS because of tight adhesions, it is reasonable to consider the largest lesion as the primary site. In addition, the immunohistochemical staining findings were consistent with a pulmonary origin. We have finally diagnosed the patient with primary lung adenocarcinoma.

With regard to disease management, some clinicians may choose ICI as the first-line treatment. However, we selected a more conservative option (a regimen of carboplatin and nanoparticle albumin-bound paclitaxel) that had a lower reported incidence rate of chemotherapy-related acute exacerbation of interstitial pneumonias [9]. This was due to the patient’s history of acute exacerbation of the indium lung that required steroid pulse therapy, which raised concerns about ICI-related pneumonitis/interstitial lung disease [7, 8]. Despite the aggressive nature of the tumor, the patient experienced the therapeutic effects and benefits of the 2-year long ICI treatment. Therefore, ICI administration could be an option even in patients with interstitial pneumonia-type indium lung.

Thus far, the patient has had a 28-year latency period from initial inhalation of indium. This period is comparable with that of asbestos for lung cancer and mesothelioma [10, 11]. A future increase in lung cancer cases in indium-exposed workers, who have substantially higher levels of sIn as seen in this case, is concerning [12].

In conclusion, we presented here the first detailed case of aggressive lung cancer that originated from indium lung; the patient benefited from a 2-year long pembrolizumab administration as second-line therapy. Industrial physicians in charge of managing indium workers and doctors who treat patients with indium lung should consider the possibility of lung cancer development.

## Data Availability

All data supporting the conclusions of this report are included within the article.

## Abbreviations

VATS: Video-assisted thoracoscopy
sIn: Serum indium levels
CT: Computed tomography
TTF-1: Thyroid transcription factor 1
FDG-PET: Fluorodeoxyglucose positron emission tomography
MRI: Magnetic resonance imaging
ICI: Immune checkpoint inhibitor
WBRT: Whole-brain radiotherapy
